# Repeatability of Pentacam HR in Keratoconus According to Two Different Scan Protocols: 25-3D Scan and 50-Cornea Fine

**DOI:** 10.3390/jcm14020439

**Published:** 2025-01-11

**Authors:** Davide Romano, Giulia Coco, Alfredo Borgia, Stefano Calza, Stephen Kaye, Kunal Gadhvi, Francesco Semeraro, Vito Romano

**Affiliations:** 1 St. Paul’s Eye Unit, Department of Corneal Diseases, Royal Liverpool University Hospital, Liverpool L7 8YA, UK; davide.romano.md@gmail.com (D.R.);; 2 Department of Clinical Science and Translational Medicine, University of Rome Tor Vergata, 00133 Rome, Italy; 3 Cornea Unit, Mons. Dimiccoli Hospital, 70051 Barletta, Italy; 4 Unit of Biostatistics and Bioinformatics, Department of Molecular and Translational Medicine, University of Brescia, 25123 Brescia, Italy; 5 Eye Clinic, Department of Medical and Surgical Specialties, Radiological Sciences and Public Health, University of Brescia, 25121 Brescia, Italy

**Keywords:** keratoconus, Pentacam, precision, cornea, repeatability, tomography, topography

## Abstract

**Background:** This study aims to evaluate the repeatability of the Pentacam HR, comparing two different measurement modes (50-cornea fine and 25-3D scan) in patients affected by keratoconus. **Methods:** Multicenter retrospective study, conducted at Eye Clinic of the ASST-Spedali Civili-University of Brescia, Italy, and St. Paul’s Eye Unit, Royal Liverpool University Hospital, United Kingdom. A total of 72 eyes from 72 patients with keratoconus underwent six consecutive measurements, three using the 25-3D scan mode and three with the 50-Cornea fine mode. Measurements were made by one single observer, using the Scheimpflug corneal tomographer camera (Pentacam HR, Oculus, Wetzlar, Germany). Repeatability was assessed using the within-subject SD (S_w_) statistic from a two-way analysis of variance. **Results:** Both measurement modes had excellent repeatability. The interclass coefficient correlation (ICC) was excellent (>0.9) in all the parameters evaluated, apart from anterior and posterior astigmatic axes and posterior astigmatism (ICC > 0.8) and index of height asymmetry (IHA) (ICC < 0.6). However, in 18 of 29 parameters, the ICC was higher in case of 25-3D scan. Repeatability limit for Kmax was 1.00D in 25-3D scan mode and 1.02D in case of 50-cornea fine. **Conclusions:** 25-3D scan may be preferable to 50-Cornea fine, in view of having slightly higher ICC in case of patients with keratoconus. Repeatability limits reported may be helpful in clinical practice for assessing the progression of keratoconus.

## 1. Introduction

Keratoconus (KC) is a bilateral and progressive ectatic disorder of cornea, characterized by asymmetrical thinning of the stroma [1,2,3]. This leads to morphological deformation of cornea, which acquires a conic shape. As a consequence, affected patients manifest visual impairment and a reduction in contrast sensitivity, due to irregular astigmatism and higher-order aberrations [4]. The clinical onset of keratoconus may occur during puberty, with an estimated prevalence of 20.6 per 1000 in males and 18.33 per 1000 in females, and may progress (continuous stromal thinning and corneal steepening) until the third to fourth decade of life [5,6].

Currently, there is no treatment for its prevention, but thanks to corneal cross linking (CXL) is possible to halt or slow its progression [7,8,9]. Therefore, is mandatory to evaluate and be sure of its progression before listing a patient for CXL [10]. To date, the natural history of KC has not been fully discovered, so apart from the known risk factors, it is not possible to determine with just one visit whether a patient will definitely experience its progression [5]. The global consensus on keratoconus has defined keratoconus progression as increased steepening of the anterior and/or posterior corneal surface or thinning, and/or an increase in the rate of corneal thickness change, from the periphery to the thinnest point [10].

Nowadays, multiple diagnostic tools have been evaluated for detecting KC and evaluating its progression, which also show good repeatability [11,12,13,14,15,16,17,18]. Among them, one of the most widely used is the Pentacam HR (high-resolution) tomographer (Oculus, Wetzlar, Germany) [15,16,18]. This device has four types of scan measurement modes: 50-Cornea fine; 100-Cornea fine; 25-3D scan; and 50-3D scan [19]. Difference between the Cornea fine and 3D scan is that, in case of 3D scan, the anterior chamber is also analyzed, whereas it is not in case of Cornea fine [19]. To date, the precision (reproducibility and repeatability) among these measurement modes has only been evaluated in cases of non-keratoconic eyes [19]. In case of keratoconus instead, precision has been evaluated using the 25-3D scan measurement mode [14,15,16,17,18].

In light of the above, our aim was to evaluate its repeatability and value limit (repeatability limit) among two different measurement modes, 50-Cornea fine and 25-3D scan, in case of keratoconus, to determine whether one type of scan should be preferred in cases of patients with KC.

## 2. Materials and Methods

Multicenter retrospective study on patients with a diagnosis of keratoconus who attended the Royal Liverpool University Hospital or the Eye Clinic of the ASST-Spedali Civili-University of Brescia in Brescia between April 2021 and March 2022. Data were collected and analyzed as aggregated and anonymous. This study followed the tenets of the Declaration of Helsinki, and the study protocol was approved by the Institutional Review Board of the University of Brescia and Royal Liverpool University Hospital.

All patients included in the study performed the Scheimpflug corneal tomographer camera (Pentacam HR, Oculus, Wetzlar, Germany, software version 1.25r15) exam during the visit, and three consecutive measurements [19] of both the 25-3D scan mode and the 50-Cornea fine scan were acquired on both eyes during the same visit. All scans were taken following the manufacturer’s instructions, under scotopic conditions without instillation of any dilating drop. Patients were instructed to remain positioned in between scans, to keep both eyes open and to look straight forward at the fixation target. Head repositioning was performed in cases of not “OK” quality scores or in cases of requests by patient.

The time between the three scans was as low as possible, with all the 6 scans generally acquired in 4 min. Randomly, either the 25-3D scan or the 50-Cornea fine scan was taken first. Exclusion criteria were previous cross-linking procedure or any other ocular surgical procedures, and history of dry eye. In case of patients wearing rigid permeable gas contact lenses, their use was discontinued four weeks prior to the examination. Data from one eye of each patient were analyzed. In case of scan quality score disparity between eyes, the eye with all scans graded as high-quality score “OK” was included. When both eyes had “OK” quality scores, the eye showing a higher Kmax value was chosen.

Investigated parameters included those which may be helpful and are more widely used to evaluate the keratoconus [16,17,18,19]: anterior maximal keratometry reading (Kmax); anterior and posterior standard keratometry (K) readings (K1 and K2); anterior and posterior average K readings (Km); anterior and posterior astigmatism (Astig) and associated axis (Axis); mean radius of anterior and posterior curvature in the 7–9mm area of the cornea (R per); minimum radius of curvature (R min); minimum corneal thickness (Pachy min); corneal thickness at corneal apex (Pachy apex); corneal volume centered on corneal apex within a dimeter of 3, 5, 7, and 10 mm (C. Vol D 3, 5, 7, 10); index of height asymmetry (IHA); index of height decentration (IHD); index of surface variance (ISV); index of vertical asymmetry (IVA); keratoconus index (KI); central keratoconus index (CKI).

In Pentacam HR software, the anterior corneal surface is abbreviated with an “F” (front), while the posterior surface with a “B” (back). Data were automatically exported from the Pentacam HR to spreadsheets. The only data which were instead manually reported in the spreadsheets were the anterior and posterior ectasia, which were taken from the “Elevation Front” and “Elevation Back” maps of the “4 Maps Refractive” report. As values of the anterior and posterior ectasia, those considered corresponded to the thinnest point of the “Corneal Thickness” map present in the “4 Maps Refractive” report.

**Figure 1:**
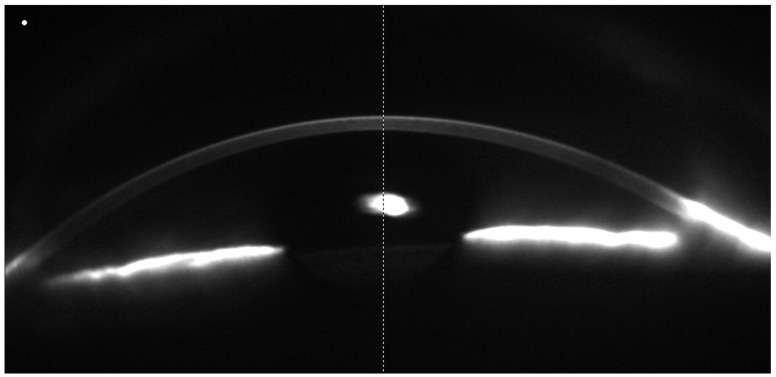
Example of a 25-3D scan. It is possible to notice that, in this type of scan, the anterior chamber is also analyzed.

**Figure 2:**
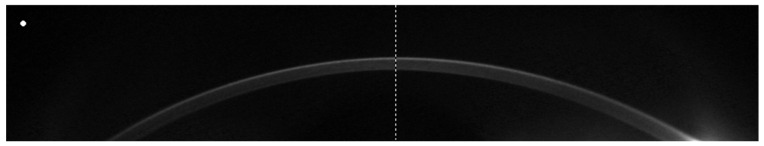
Example of a 50-Cornea fine scan. It is possible to notice that, in this type of scan, only the cornea is analyzed.

Figure 1 and Figure 2 report an example of a 25-3D scan (Figure 1) and a 50-Cornea scan (Figure 2), respectively.

### Statistical Analysis

Summary statistics were used to describe the participants’ demographics and measurements readings as means or as percentages for categorical variables.

The repeatability of each parameter, quantified by the Pentacam HR for each mode, was assessed using the intraclass correlation coefficient (ICC). Repeatability refers to the variability in repeated measurements by one method and one observer when all other factors are assumed constant [20]. The ICC was estimated using a two-way random effects model (both patient and evaluation, i.e., time, were considered as random effects). Given very high ICC values for each method, precision was then also tested, following the recommendations of the British Standards Institute and the International Organization for Standardization. Repeatability estimates were expressed in terms of standard deviations (SD). One-way analysis of variance (ANOVA) was performed to determine the repeatability of measurements, which equals the within-subjects SD (S) for repeated measurements with the same observer.

The 95% confidence interval (95% CI) was calculated as the repeatability limits (S_r_), reported as 1.96 √2x S_w_. S_w_ and S_r_ were calculated for the repeated measurements with the two scan modes (25-3D scan and 50-Cornea Fine). More precise measurements correspond to lower S_r_ values. Statistical analysis was performed using STATA, version 13.0 (StataCorp LLC, College Station, TX, USA).

## 3. Results

Seventy-two eyes of seventy-two patients with a mean age of 29.4 ± 9.5 years (68% males) were scanned three consecutive times with both the 25-3D scan and 50-Cornea fine scan modes, as described in the Methods section. The right eye was scanned in 34 patients (47%).

The mean readings with the 25-3D scan and 50-Cornea fine scan modes were 54.1D for Kmax, 44.6D for anterior K1, 47.7D and 47.6D for anterior K2, 46.1D and 46.0D for anterior Km, 3.1D for astigmatism and 87.7° and 90.6° for its axis, −6.6D for posterior K1, −7.3D for posterior K2, −6.9D for posterior Km, 0.7D for posterior astigmatism and 98.1° and 97.4° for its axis, 468.9um and 467.4um for Pachy min, 28.2 and 28.0 for anterior ectasia, 58.6 for posterior ectasia, 85.0 and 85.4 for the ISV, and 1.0 and 0.98 for the IVA. Mean readings are shown in Table 1.

The ICC with relative 95% CI for each measurement, analyzed with both 25-3D scan and 50-Cornea fine modes, are shown in Table 2.

Overall, both scans showed excellent repeatability, with most ICC > 0.9. Lower repeatability was found in anterior and posterior astigmatic axes and posterior astigmatism; however, these still showed good values (>0.8) and IHA (ICC < 0.6).

The repeatability (S_w_) and repeatability limits (S_r_) are reported in Table 3.

## 4. Discussion

Reliable and precise evaluation of corneal measurements is essential for the diagnosis, follow-up, and management of patients with keratoconus [10].

One of the most common instruments used in clinical practice to evaluate cornea in eyes with keratoconus is the Pentacam HR tomographer, whose precision has already been evaluated in the literature, but using only one measurement mode, the 25-3D scan [14,15,16,17,18].

However, the Pentacam HR has two acquisition protocols, 3D scan and Cornea fine, and four measurement modes, which are listed as follows: 25-3D scan and 50-Cornea fine, which have the same acquisition time of 1 s, and 50-3D scan and 100-Cornea fine, which have the same acquisition time of 2 s [19].

Differences between the 3D scan and Cornea fine are in the evaluation of the anterior chamber, which is performed by the first, but not by the second [19]. However, the second acquires the double of cornea sagittal sections at the same time. The repeatability of these measurement modes, among a wide range of parameters, has only been reported in healthy cornea to date, so the aim of our study was to evaluate whether or not one of the two measurement modes, 25-3D scan and 50-Cornea fine, had higher repeatability and, subsequently it should be preferred when using the Pentacam HR tomographer in patients with keratoconus.

The other two measurement modes, 50-3D scan and 100-Cornea fine, were not evaluated, in view of the longer acquisition time is reported to cause a decline in precision, probably related to less patient compliance with a subsequent greater chance of eye movements.

The results of our study showed that both automated measurement modes, 25-3D scan and 50-Cornea fine, had excellent repeatability, with an ICC > 0.9 in all the evaluated parameters, apart from anterior and posterior astigmatic axes and posterior astigmatism (ICC > 0.8) and index of height asymmetry (IHA) (ICC < 0.6).

However, in a higher number of parameters, the ICC was higher in case of 25-3D scan, while parameters in which ICC was higher in 50-Cornea fine were, instead, especially those relative to the cornea volume (evaluation of corneal thinnest point, cornea pachymetry at cornea apex and cornea volume at 3, 4, 7, and 10 mm). Another parameter which had higher ICC in 50-Cornea fine was relative to axis of the anterior astigmatism.

Its higher repeatability suggests that, in case of planning to implant intrastromal corneal segment rings, in which the astigmatism axis is important, the 50-Cornea fine measurement mode may be preferred, although the difference in the front axis is minimal (2.9° accordingly our results), and its clinical implications have to be addressed with specific studies with intrastromal rings [21,22].

An additional advantage of 25-3D scan is the evaluation of the anterior chamber, which is lacking in 50-Cornea fine, but may be helpful in patients with keratoconus in cases of the co-existing presence of glaucoma, for pre-operative examination or glaucoma management [23].

Surprisingly, according to these results, the double of sagittal scans of the cornea, provided by the Cornea fine measurement mode, does not guarantee a higher repeatability. This could be explained by the view that, in keratoconus, the corneal morphology is less homogeneous. Consequently, a higher number of scans may result in a greater variability in the data acquired by the Pentacam, leading to a slightly reduced repeatability for individual parameters. In contrast, in healthy eyes, the repeatability of the 50-Cornea fine mode was notably higher compared to the 25-3D scan mode [19].

Regarding the repeatability limit, this study provided reference values of an extensive number of relevant parameters. Repeatability implies that, to define the progression for the parameter evaluated, its change should be greater that the repeatability limit value [20].

Compared with repeatability limits in healthy cornea, reported by McAlinden et al., in our results, the variability is higher regardless of the measurement mode used [19].

The problem is that to date, there is still a lack of consensus on the quantitative values that should be used to define ectasia progression. The Global Consensus on Keratoconus and Ectatic Diseases defined the parameters that should be evaluated, but no numerical threshold reference values [10].

If the threshold value of 1D in Kmax is often considered as a cut-off reference to confirm progression and list a patient for CXL, the risk of overtreatment would be greater. Indeed, this value of 1D in Kmax was based on the results of the Kmax repeatability limit of 0.80D for the 25-3D scan, and 0.55D for 50-Cornea fine [19].

Instead, our results showed that the repeatability limit for Kmax was 1.00D in 25-3D scan mode and 1.02D in 50-Cornea fine mode, which is almost the double the value for the same protocol in healthy eyes [19]. It could be argued that the repeatability limit of 1.00D and 1.02D for Kmax is consistent with the previously mentioned cut-off of 1.00D for keratoconus progression, but we had three acquisitions for each measurement mode, and only with the high-quality score of “OK”.

Our study has several limitations. Differences in ICC and repeatability between these two measurement modes were not evaluated for other parameters due to the impossibility to automatically export all the data, or the lack of them. The number of eyes included was limited to 72 from 72 patients, with a prevalence of males and without any pediatric patients. Additional limitations are the lack of a control group, and sub-analysis in cases of contact lens wearers and naïve patients. Further studies are needed to address these limitations in order to corroborate our findings, as well as to assess if the 50-Cornea fine mode should be preferred in cases of planning corneal stromal rings.

## 5. Conclusions

In conclusion, both measurement modes, 25-3D scan and 50-Cornea fine, have good repeatability among a wide range of parameters, thus confirming that both are useful in clinical and research settings not only in healthy eyes, but also in eyes with keratoconus. In view of these results, the 25-3D scan measurement mode should be preferred over the 50-Cornea fine in routine clinical practice.

## Figures and Tables

**Table 1:** Mean readings of 25-3D scan and 50-Cornea fine.

Measurement (Unit of Measurement)	25-3D Scan	50-Cornea Fine
Kmax (D)	54.1	54.1
Pachy min (µm)	468.9	467.4
Anterior ectasia (µm)	28.2	28
Posterior ectasia (µm)	58.6	58.6
K1 F (D)	44.6	44.6
K2 F (D)	47.7	47.6
Km F (D)	46.1	46
Axis F	87.7°	90.6°
Astigm F (D)	3.1	3.1
K1 B (D)	−6.6	−6.6
K2 B (D)	−7.3	−7.3
Km B (D)	−6.9	−6.9
Axis B	98.1°	97.4°
Astigm B (D)	0.7	0.7
ISV	85	85.4
IVA	1	0.98

Kmax: anterior maximal keratometry reading; Pachy min: minimum corneal thickness; K1 and K2: anterior and posterior standard keratometry (K) readings; Km: anterior and posterior average K reading; Astigm: anterior and posterior astigmatism; Axis: astigmatism axis; ISV: index of surface variance; IVA: index of vertical asymmetry.

**Table 2:** Intraclass correlation coefficient (ICC) with relative 95% CI.

Measurement	25-3D Scan		50-Cornea Fine	
	ICC	95% CI	ICC	95% CI
Kmax (D)	0.9957 ^#^	0.9937–0.9972	0.9956	0.9934–0.9971
Pachy min	0.9873	0.9813–0.9916	0.9912 ^#^	0.9871–0.9942
Anterior ectasia	0.9888 ^#^*	0.9836–0.9926	0.9511	0.9286–0.9676
Posterior ectasia	0.9662 ^#^	0.9506–0.9776	0.9318	0.9011–0.9546
K1 F	0.9965 ^#^	0.9931–0.9969	0.9933	0.9901–0.9956
K2 F	0.9948 ^#^	0.9924–0.9966	0.9921	0.9883–0.9948
Km F	0.9966 ^#^	0.9949–0.9977	0.9940	0.9911–0.9960
Axis F	0.7627	0.6740–0.8350	0.8017 ^#^	0.7244–0.8633
Astigm F	0.9560	0.9358–0.9707	0.9626 ^#^	0.9454–0.9752
R per F (mm)	0.9964 ^#^	0.9946–0.9976	0.9935	0.9904–0.9957
R min (mm)	0.9957 ^#^	0.9936–0.9971	0.9948	0.9923–0.9966
K1 B	0.9888 ^#^	0.9835–0.9926	0.9876	0.9818–0.9918
K2 B	0.9893 ^#^	0.9843–0.9930	0.9798	0.9704–0.9867
Km B	0.9933 ^#^	0.9901–0.9956	0.9890	0.9839–0.9928
Axis B	0.8617 ^#^	0.8043–0.9061	0.8403	0.7754–0.8909
Astigmatism B	0.8903 ^#^	0.8433–0.9260	0.8751	0.8225–0.9154
R per B	0.9794 ^#^	0.9697–0.9864	0.9607	0.9427–0.9739
R min B	0.9684	0.9538–0.9791	0.9768 ^#^	0.9659–0.9846
Pachy apex	0.9885	0.9831–0.9924	0.9919 ^#^	0.9881–0.9947
C. Vol D 3 mm	0.9763	0.9652–0.9843	0.9872 ^#^	0.9812–0.9916
C. Vol D 5 mm	0.9855	0.9786–0.9904	0.9895 ^#^	0.9845–0.9931
C. Vol D 7 mm	0.9837	0.9760–0.9892	0.9875 ^#^	0.9815–0.9917
C. Vol D 10 mm	0.9799	0.9705–0.9867	0.9820 ^#^	0.9736–0.9881
ISV	0.9952 ^#^	0.9930–0.9969	0.9951	0.9927–0.9968
IVA	0.9891 ^#^	0.9839–0.9928	0.9886	0.9832–0.9925
KI	0.9908	0.9864–0.9939	0.9917 ^#^	0.9878–0.9946
CKI	0.9905 ^#^	0.9860–0.9937	0.9891	0.9840–0.9928
IHA	0.5881 ^#^	0.4619–0.7009	0.4953	0.3575–0.6247
IHD	0.9837	0.9760–0.9892	0.9885 ^#^	0.9831–0.9924

^#^: Higher ICC coefficient; *: 95% CI of the 25-3D scan and 50-Cornea fine scan modes do not overlap; ICC: intraclass correlation coefficient; Kmax: anterior maximal keratometry reading; K1 and K2: anterior and posterior standard keratometry (K) readings; Km: anterior and posterior average K readings; Astigm: anterior and posterior astigmatism; Axis: astigmatism axis; R per: mean radius of anterior and posterior curvature in the 7–9 mm area of the cornea; R min: minimum radius of curvature; Pachy min: minimum corneal thickness; Pachy apex: corneal thickness at cornea apex; C. Vol D 3 mm, 5 mm, 7 mm, 10 mm: corneal volume centered on corneal apex within a diameter of 3 mm, 5 mm, 7 mm, and 10 mm; ISV: index of surface variance; IVA: index of vertical asymmetry; KI: keratoconus index; CKI: central keratoconus index; IHA: index of height asymmetry; IHD: index of height decentration.

**Table 3:** Repeatability (S_w_) and repeatability limits (S_r_).

Measurement (Unit of Measurement)	25-3D Scan	50-Cornea Fine
Kmax (D)	0.36 (1.00)	0.37 (1.02)
Pachy min (µm)	4.26 (11.81)	3.57 (9.88)
Anterior ectasia (µm)	1.40 (3.87)	3.03 (8.38)
Posterior ectasia (µm)	3.93 (10.87)	6.19 (17.15)
K1 F (D)	0.27 (0.74)	0.33 (0.91)
K2 F (D)	0.31 (0.85)	0.38 (1.05)
Km F (D)	0.24 (0.66)	0.31 (0.87)
Axis F (D)	29.22 (80.95)	25.95 (71.87)
Astigm F (D)	0.35 (0.96)	0.33 (0.90)
R per F (mm)	0.02 (0.05)	0.02 (0.07)
R min (mm)	0.04 (0.11)	0.04 (0.12)
K1 B (D)	0.08 (0.23)	0.09 (0.25)
K2 B (D)	0.09 (0.24)	0.12 (0.33)
Km B (D)	0.06 (0.18)	0.08 (0.24)
Axis B (degree)	24.09 (66.74)	25.00 (69.26)
Astigmatism B (D)	0.11 (0.31)	0.12 (0.34)
R per B	0.04 (0.10)	0.05 (0.15)
R min B	0.10 (0.27)	0.08 (0.23)
Pachy apex (µm)	4.29 (11.89)	3.67 (10.18)
C. Vol D 3 mm	0.04 (0.11)	0.03 (0.08)
C. Vol. D 5 mm	0.08 (0.23)	0.07 (0.19)
C. Vol D 7 mm	0.17 (0.47)	0.15 (0.42)
C. Vol D 10 mm	0.45 (1.24)	0.43 (1.18)
ISV	2.25 (6.22)	2.32 (6.43)
IVA	0.04 (0.12)	0.05 (0.13)
KI	0.01 (0.03)	0.01 (0.03)
CKI	0.01 (0.01)	0.01 (0.02)
IHA	14.45 (40.02)	15.50 (42.94)
IHD	0.02 (0.06)	0.01 (0.02)

Kmax: anterior maximal keratometry reading; K1 and K2: anterior and posterior standard keratometry (K) readings; Km: anterior and posterior average K reading; Astigm: anterior and posterior astigmatism; Axis: astigmatism axis; R per: mean radius of anterior and posterior curvature in the 7–9 mm area of the cornea; R min: minimum radius of curvature; Pachy min: minimum corneal thickness; Pachy apex: corneal thickness at cornea apex; C. Vol D 3 mm, 5 mm, 7 mm, 10 mm: corneal volume centered on corneal apex within a diameter of 3 mm, 5 mm, 7 mm, and 10 mm; ISV: index of surface variance; IVA: index of vertical asymmetry; KI: keratoconus index; CKI: central keratoconus index; IHA: index of height asymmetry; IHD: index of height decentration.

## Data Availability

The original contributions presented in this study are included in the article. Further inquiries can be directed to the corresponding author.

## References

[1] Rabinowitz Y.S. (1998). Keratoconus. Surv. Ophthalmol..

[2] Padmanabhan P., Lopes B.T., Eliasy A.M., Abass A., Vinciguerra R., Vinciguerra P., Ambrósio R.J., Elsheikh A. (2022). Evaluation of corneal biomechanical behavior in vivo for healthy and keratoconic eyes using the stress–strain index. J. Cataract. Refract. Surg..

[3] Herber R., Hasanli A., Lenk J., Vinciguerra R., Terai N., Pillunat L.E., Raiskup F. (2022). Evaluation of Corneal Biomechanical Indices in Distinguishing Between Normal, Very Asymmetric, and Bilateral Keratoconic Eyes. J. Refract. Surg..

[4] Santodomingo-Rubido J., Carracedo G., Suzaki A., Villa-Collar C., Vincent S.J., Wolffsohn J.S. (2022). Keratoconus: An updated review. Contact Lens Anterior Eye.

[5] Hashemi H., Heydarian S., Hooshmand E., Saatchi M., Yekta A.A., Aghamirsalim M., Valadkhan M., Mortazavi M., Hashemi A., Khabazkhoob M. (2020). The Prevalence and Risk Factors for Keratoconus: A Systematic Review and Meta-Analysis. Cornea.

[6] Gokul A., Patel D.V., Watters G.A., McGhee C.N.J. (2016). The natural history of corneal topographic progression of keratoconus after age 30 years in non-contact lens wearers. Br. J. Ophthalmol..

[7] Meiri Z., Keren S., Rosenblatt A., Sarig T., Shenhav L., Varssano D. (2016). Efficacy of Corneal Collagen Cross-Linking for the Treatment of Keratoconus. Cornea.

[8] Borchert G.A., Kandel H., Watson S.L. (2023). Epithelium-on versus epithelium-off corneal collagen crosslinking for keratoconus: A systematic review and meta-analysis. Graefe’s Arch. Clin. Exp. Ophthalmol..

[9] Karam M.M., Alsaif A.M., Aldubaikhi A.M., Aljebreen M.M., Alazaz R.M., Alkhowaiter N., Almudhaiyan T., Aljassar F.F. (2022). Accelerated Corneal Collagen Cross-Linking Protocols for Progressive Keratoconus: Systematic Review and Meta-analysis. Cornea.

[10] Gomes J.A.P., Tan D., Rapuano C.J., Belin M.W., Ambrósio R., Guell J.L., Malecaze F., Nishida K., Sangwan V.S. (2015). Group of Panelists for the Global Delphi Panel Panel of Keratoconus and Ectatic Disease Global Consensus on Keratoconus and Ectatic Diseases. Cornea.

[11] Seiler T.G., Mueller M., Baiao T.M. (2022). Repeatability and Comparison of Corneal Tomography in Mild to Severe Keratoconus Between the Anterior Segment OCT MS-39 and Pentacam HR. J. Refract. Surg..

[12] Hashemi H., Khabazkhoob M., Pakzad R., Bakhshi S., Ostadimoghaddam H., Asaharlous A., Yekta R., Aghamirsalim M., Yekta A. (2019). Pentacam Accuracy in Discriminating Keratoconus From Normal Corneas: A Diagnostic Evaluation Study. Eye Contact Lens Sci. Clin. Pr..

[13] Lopes B., Padmanabhan P., Zhang H., Abass A., Eliasy A., Bandeira F., Bao F., Bühren J., Elmassry A., Faria-Correia F. (2021). Clinical Validation of the Automated Characterization of Cone Size and Center in Keratoconic Corneas. J. Refract. Surg..

[14] Kreps E.O., Jimenez-Garcia M., Issarti I., Claerhout I., Koppen C., Rozema J.J. (2020). Repeatability of the Pentacam HR in Various Grades of Keratoconus. Arch. Ophthalmol..

[15] Eguileor B.d.L., Argaluza J.E., Zubizarreta J.I.P., Carro A.S., Ecenarro J.E. (2017). Evaluation of the Reliability and Repeatability of Scheimpflug System Measurement in Keratoconus. Cornea.

[16] Kosekahya P., Koc M., Caglayan M., Kiziltoprak H., Atilgan C.U., Yilmazbas P. (2018). Repeatability and reliability of ectasia display and topometric indices with the Scheimpflug system in normal and keratoconic eyes. J. Cataract. Refract. Surg..

[17] Gustafsson I., Bergström A., Myers A.C., Ivarsen A., Hjortdal J. (2020). Association between keratoconus disease severity and repeatability in measurements of parameters for the assessment of progressive disease. PLoS ONE.

[18] Hashemi K., Guber I., Bergin C., Majo F. (2015). Reduced Precision of the Pentacam HR in Eyes with Mild to Moderate Keratoconus. Ophthalmology.

[19] McAlinden C., Khadka J., Pesudovs K. (2011). A Comprehensive Evaluation of the Precision (Repeatability and Reproducibility) of the Oculus Pentacam HR. Investig. Opthalmol. Vis. Sci..

[20] McAlinden C., Khadka J., Pesudovs K. (2011). Statistical methods for conducting agreement (comparison of clinical tests) and precision (repeatability or reproducibility) studies in optometry and ophthalmology. Ophthalmic Physiol. Opt..

[21] Jacob S., Patel S.R., Agarwal A., Ramalingam A., Saijimol A.I., Raj J.M. (2018). Corneal Allogenic Intrastromal Ring Segments (CAIRS) Combined With Corneal Cross-linking for Keratoconus. J. Refract. Surg..

[22] Cueto L.F.-V., Lisa C., Poo-López A., Madrid-Costa D., Merayo-Lloves J., Alfonso J.F. (2016). Intrastromal Corneal Ring Segment Implantation in 409 Paracentral Keratoconic Eyes. Cornea.

[23] Hashemi H., Yekta A., Yazdani N., Ostadimoghaddam H., Khabazkhoob M. (2020). Comparison of Anterior Chamber Depth between Normal and Keratoconic Eyes. J. Curr. Ophthalmol..

